# Electrochemical Skin Conductance Correlates with Skin Nerve Fiber Density

**DOI:** 10.3389/fnagi.2016.00199

**Published:** 2016-08-24

**Authors:** Peter Novak

**Affiliations:** Autonomic Laboratory, Department of Neurology, Brigham and Women's Faulkner Hospital, Harvard Medical SchoolBoston, MA, USA

**Keywords:** electrochemical skin conductance, sweat gland nerve fiber density, small fiber neuropathy, skin biopsy, epidermal nerve fiber density

## Abstract

**Purpose:** Electrochemical skin conductance (ESC) using reverse iontophoresis and chronoamperometry has been used to evaluate abnormal function of small fibers. How ESC correlates with loss of small fibers in skin is unclear.

**Methods:** This was a prospective, blinded study. The primary outcome measure was the correlation between ESC at the feet and results of skin biopsies including epidermal nerve fiber density (ENFD) and sweat gland nerve fiber density (SGNFD) at the distal leg. ESC, ENFD, and SGNFD data were normalized by adjusting for weight. The secondary outcome measures were the correlation between ESC and the following variables: quantitative sudomotor axon reflex test (QSART) and symptom scales (neuropathy, pain and autonomic).

**Results:** Eighty-one patients (mean ± sd): age = 53.3 ± 17.3, men/women = 25/56 were enrolled in the study. ESC was reduced in subjects with abnormally low ENFD (ENFD normal/abnormal, ESC = 1.17 ± 0.27/0.87 ± 0.34 μSiemens/kg, *p* < 0.0008) and abnormally low SGNFD (SGNFD normal/abnormal ESC = 1.09 ± 0.34/0.78 ± 0.3 μSiemens/kg, *p* < 0.0003). ESC correlated with ENFD (ρ = 0.73, *p* = 0.0001) and SGNFD (ρ = 0.64, *p* = 0.0001). ESC did not correlate with symptom scales.

**Conclusion:** ESC is diminished in subjects who have a reduced number of small fibers in the skin and the ESC reduction is proportional to ENFD and SGNFD. ESC can be useful in detecting loss of small nerve fibers.

## Introduction

Small fiber neuropathy (SFN) is common, affecting millions of people worldwide, and may be associated with considerable disability (England et al., 2009; Lauria et al., 2010; Hovaguimian and Gibbons, 2011; Hoeijmakers et al., 2012). Small nerve fibers, thinly myelinated Aδ-fibers and unmyelinated C-fibers, can be separated into autonomic (efferent) or sensory (afferent). Their dysfunction can result in dysautonomia due to autonomic SFN, sensory symptoms due to sensory SFN, or a combination of both due to mixed sensory and small fiber SFN. Mixed SFN is the most common form (Novak et al., 2001; Devigili et al., 2008; Hovaguimian and Gibbons, 2011). Idiopathic SFN, in which no cause can be identified, represents about 50% of all SFN cases. The remaining secondary SFNs are associated with multiple disorders including impaired glucose tolerance, metabolic syndrome, thyroid dysfunction, sarcoidosis, vitamin B12 deficiency, HIV, autoimmune syndromes, neurotoxic medications including chemotherapeutic and antiretroviral agents, celiac disease, paraneoplastic syndromes, alcohol abuse, elevated triglycerides and paraproteinemias (England et al., 2009; Lauria et al., 2010; Hovaguimian and Gibbons, 2011; Hoeijmakers et al., 2012).

Although there is wide heterogeneity of SFN causes, the available diagnostic tools detect either abnormal nerve function (functional methods) or loss of axons (morphological methods), irrespective of the underlying etiology. The morphological methods include skin biopsies for assessment of sensory fibers using epidermal nerve fiber density (ENFD) and sudomotor fibers using sweat gland nerve fiber density (SGNFD). Among the objective functional tests, the quantitative sudomotor axon reflex test (QSART) evaluating postganglionic sudomotor function is most widely used (Hovaguimian and Gibbons, 2011). A recent addition is the measurement of the electrochemical skin conductance (ESC) using Sudoscan technology (Impeto Medical, Paris, France) (Casellini et al., 2013; Smith et al., 2014). The Sudoscan device applies a small electrical stimulus to the skin of the hands and palms; ESC is the derivative current produced by sweat chloride ions (reverse iontophoresis) in response to the applied stimulus, and has been found to be useful in the diagnosis of diabetic and non-diabetic SFN (Casellini et al., 2013; Smith et al., 2014). Distinct advantages of ESC are safety, ease to use, speed and its noninvasive character. Reduced ESC may reflect loss of small fibers; however, the direct comparison of ESC with underlying pathology is lacking. In this study, a direct functional-pathological correlation was performed where ESC was compared to ENFD and SGNFD.

## Methods

### Study design

This was a prospective, blinded study. The study was approved by the Institutional Review Board of the University of Massachusetts Medical School and all subjects signed an informed consent.

### Participants

The study participants included subjects who were referred for evaluation of SFN to the tertiary care setting at the University of Massachusetts Medical School, Autonomic Laboratory in 2015. Recruitment was based on completing the SFN evaluations. Inclusion criteria were patients older than 17 years who completed evaluation for SFN at the autonomic laboratory, had available electronic medical records and provided informed consent. Electronic records were reviewed for secondary causes of loss of small fibers including diabetes, borderline diabetes, large fiber neuropathy, Parkinson's disease, atypical parkinsonism, known history of heavy alcohol exposure, B12 deficiency, folate deficiency, thyroid disease, hepatitis C, HIV infection, exposure to chemotherapy, use of neurotoxic medication, cancer, any comorbid condition or use of medication reported to be associated with small fiber neuropathy. Hemoglobin A1c (HbA1c), B12, folate, and TSH levels were also collected for further analysis.

Patient's records were also reviewed for presence and character of neuropathic pain (spontaneous, evoked, burning, aching, stabbing, allodynic), numbness, tingling or pins and needles sensation as well as distribution of sensory symptoms (length or non-length dependent). The records were screened for autonomic symptoms including postural dizziness and hypotension, sphincter problems, abnormal sweating, and gastrointestinal symptoms. Neurological examinations were reviewed to identify abnormalities in proprioceptive evaluation, muscle strength or deep tendon reflexes that may indicate large fiber involvement. Particular emphasis was placed on identifying signs of SFN including pinprick and thermal sensory loss, allodynia and hyperalgesia (Devigili et al., 2008).

### Evaluation of sensory and autonomic symptoms

This evaluation was done at the autonomic laboratory as part of the SFN evaluation. Sensory evaluations included self-report of pain intensity using a 0 to 10 numerical rating scale (Cook et al., 2013), the Neuropathy Pain Symptom Inventory (Bouhassira et al., 2004) and the Neuropathy Total Symptom Score-6 (Bastyr et al., 2005). Autonomic symptoms were assessed using the Survey of Autonomic Symptoms (Zilliox et al., 2011).

### Skin biopsies

Skin biopsies were performed for assessment of ENFD and SGNFD. Skin biopsies were performed according to the recommended standards (McArthur et al., 1998; Lauria et al., 2010). Briefly, two skin biopsy samples were obtained, one from the proximal thigh 20 cm distal to the iliac spine and the other at the calf (10 cm above the lateral malleolus) using a 3-mm circular disposable punch tool. Samples were immediately transferred into 2% paraformaldehyde-lysine-periodic acid fixative and transported overnight to the laboratory using cold packs to maintain cooling. Tissue processing was conducted at a commercial laboratory (Therapath, New York, NY). Therapath is a CLIA-certified laboratory and is accredited by the College of American Pathologists. Samples were immunoperoxidase-stained for the pan-axonal marker PGP 9.5. Linear ENFD was determined using bright light microscopy according to the guidelines of the European Federation of the Neurological Societies (Lauria et al., 2010). The density was calculated in at least 3 sections as the number of nerve fibers per length of the section (density/mm). Only single fibers crossing the dermal-epidermal junctions were counted while secondary branching was excluded from the analysis.

SGNFD was determined using the same tissue sections stained for PGP 9.5. Images of one or two sweat glands were captured using an Olympus photomicrograph. The digital images were analyzed according to the method of (Gibbons et al., 2009). All sweat gland nerve fiber counts were done by one board certified neuropathologist who was blinded to the ESC results. The SGNFD intra-rater variability was 8.4 ± 9.6% (mean and standard deviation for 48 analyzed sweat glands during the last 1 year period) and the inter-rater variability was 10.2 ± 8.4% for two observers within the same year (Dr. Hays, Therapath, personal communication).

The limits of normality for ENFD at the thigh are 6.2/8.3 (men/women) fibers per millimeter of epidermal length as determined by Therapath. The University of Massachusetts' limits of normal ENFD (fibers per millimeter of epidermal length) at the calf are equal to 9.5–0.075^*^age for men and 11.1–0.08^*^age for women (Novak, 2015). The limits of normality for SGN FD at the thigh and the calf are 37.8 and 36.5 fibers per millimeter as determined by Therapath.

### Autonomic testing

Autonomic function testing was performed following established standards that have been described in detail elsewhere (Novak, 2011, 2015). Cardiovascular reflex tests included deep breathing, the Valsalva maneuver and tilt test. Postganglionic sudomotor functions were assessed by QSART in the forearm, proximal leg, distal leg, and foot, using the Q-Sweat machine (WR Medical Electronics, Stillwater, MN).

### Sudomotor function testing using ESC

ESC assessment was performed using the Sudoscan device (Impeto Medical, Paris, France) (Casellini et al., 2013; Smith et al., 2014). Subjects were asked to place both palms and soles on large area stainless-steel electrodes during the 3-min scan. The ESC data acquisition was performed in the standing position. A low direct current (DC) voltage (<4 V) is applied incrementally to the electrodes (chronoamperometry), generating a current (around 0.2 mA) proportional to the chloride ions extracted from the skin (reverse iontophoresis). The ratio of the current generated by the reaction of the chloride ions with the electrodes and the constant DC stimulus applied is calculated for each foot and hand and expressed as the ESC measured in microSiemens (μS).

### Definition of SFN

Devigili et al. (2008) defined SFN by requiring the presence of abnormalities in at least two of the following 3 examinations: (1) clinical signs suggestive of SFN (pinprick or thermal sensory loss, allodynia and hyperalgesia); (2) abnormal Quantitative Sensory Testing (QST) and (3) reduced ENFD. The combination of clinical signs and ENFD provides the highest diagnostic accuracy (Devigili et al., 2008). These criteria use only sensory markers and SFN also affects autonomic fibers (Novak et al., 2001; Devigili et al., 2008; Hovaguimian and Gibbons, 2011). The diagnostic yield of Devigili's criteria can be increased by adding sudomotor evaluation (Low et al., 2006; Thaisetthawatkul et al., 2013, 2014). Therefore SFN was defined by the presence of all of the following: (1) clinical signs suggestive of SFN and (2) at least one abnormal test out of the following objective tests: ENFD, SGNFD, QSART, or sympathetic adrenergic evaluation.

The SFN patients were further stratified into idiopathic SFN, SFN secondary to other causes and SFN combined with large fiber neuropathy.

### Outcome measures

The primary outcome measure was the correlation between ESC at the feet and skin biopsies (ENFD and SGNFD) at the calf. The secondary outcome measures were the correlations between feet ESC and other continuous variables (QSART and symptom scales).

## Statistical analysis

**Figure 1:**
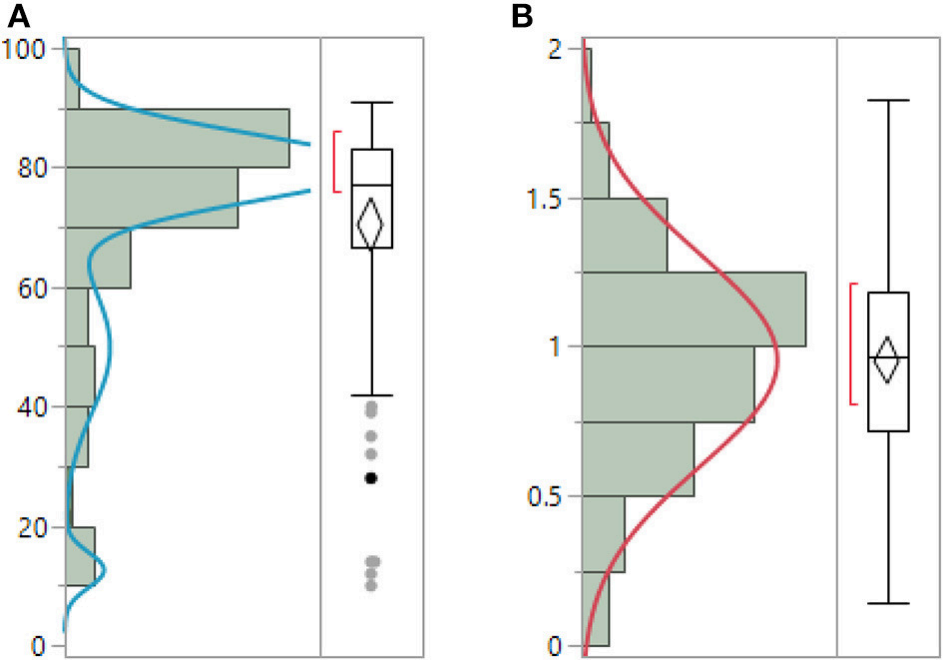
**Unadjusted raw ESC data (A) have non-normal distribution and can be best-fitted by mixture of 3 normal distributions**. ESC data adjusted for weight **(B)** have normal distribution. (μ = 0.95, σ = 0.35).

ENFD, SGNFD, and feet ESC have non-normal distribution. Both ENFD and SGNFD were best approximated by a mixture of 2 normal distributions, while ESC by a mixture of 3 normal distributions. ENFD, SGNFD, and ESC data were normalized by adjusting for weight [e.g., normalized ESC data = raw ESC/weight (kg)] (Figure 1). The adjustment resulted in normal distribution for ESC, exponential distribution for ENFD and Weibull distribution for SGNFD. Adjusted data were used for correlation analysis if not indicated otherwise.

Feet ESC in subjects who had abnormal biopsies were compared to subjects with normal biopsies using non-parametric Wilcoxon test.

The relationships between continuous variables were obtained using Spearman's rank correlation (ρ) coefficient. Least squares (LS) models were used to evaluate the relationships between skin biopsies and ESC as a dependent variable with age and sex as model effect. LS models were calculated separately for ENFD and SGNFD to minimize the effects of multiple comparisons. Receiver operating characteristics (ROC) curves were calculated to compare the diagnostic utility of ESC and QSART using ENFD and SGNFD as a reference.

### Power calculations

Since multiple comparisons have been performed for correlations calculations, the significance was adjusted using Bonferroni corrections. For a hypothesis with a desired α = 0.05 and 15 comparisons, the Bonferroni-corrected α became equal to 0.003 (obtained by 0.05/15) which is required for the tests to be significant. All statistical analysis were performed using JMP 12.0 (Cary, NC) statistical software.

## Results

Eighty-two consecutive patients evaluated for SFN were enrolled in the study [(mean ± sd): age = 53.3 ± 17.3 years, men/woman = 25/56, BMI = 27.9 ± 7.0 kg/m^2^]. None of our subject experienced any complications of skin biopsies, autonomic testing or any side effect associated with use of the Sudoscan device. ENFD was obtained in 82 subjects, SGNFD in 70 subjects. In 12 subjects no sweat glands were identified in the skin biopsies. ESC data from one subject were invalid due motion artifact and 81 subjects were used for statistical analysis.

SFN was identified in all subjects. ENFD was abnormal in 58 subjects, SGNFD in 25 subjects, QSART in 63 subjects, and 71 subjects had abnormal sympathetic adrenergic score that evaluates blood pressure responses to the Valsalva maneuver and tilt table.

The most common comorbidities were hypertension (*n* = 14) and migraine (12). Disorders associated with SFN were identified in 33 subjects (*n*), including diabetes (9), impaired glucose tolerance (2), alcoholic neuropathy (2), thyroid disorder (4), B12 deficiency (2), Parkinson disease (2), elevated triglycerides (2), metabolic syndrome (4), Sjögren syndrome (1), autoimmune SFN (1), monoclonal gammopathy (2), end-stage renal disease (1) and exposure to chemotherapy (1). Four patients in the mixed autonomic and sensory SFN also had a large fiber mixed sensory-motor length dependent neuropathy confirmed by abnormal nerve conduction studies (NCS).

Idiopathic SFN, was identified in 48 (59%) patients with the most disabling symptom being a distal burning sensation (14), orthostatic symptoms (22), sphincter problems (6) and cold or heat intolerance (4).

**Figure 2:**
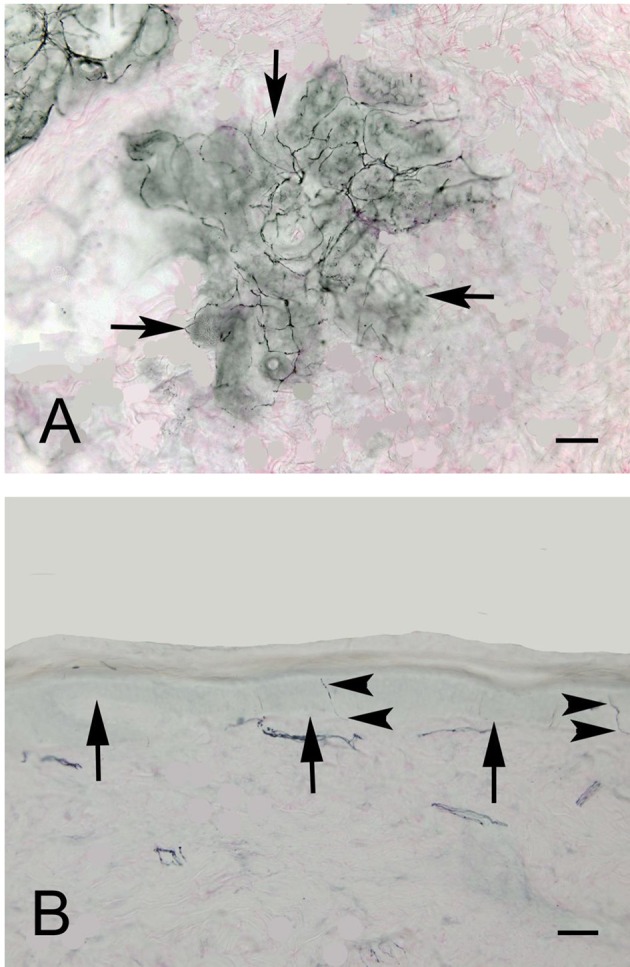
**Representative examples of SGNFD (A) and ENFD (B)**. Tissue processing for SGNFD and ENFD was done using bright-field immunohistochemistry with anti-9.5 antibodies. The tissue sections were counterstained with eosin. In Figure 1A, the arrows point to a sweat gland. In Figure 1B, the arrows point to the epidermal-dermal junction and the arrowheads point to epidermal nerve fibers. Bar = 50 microns. SGNFD, sweat gland nerve fiber density; ENFD, epidermal nerve fiber density.

**Figure 3:**
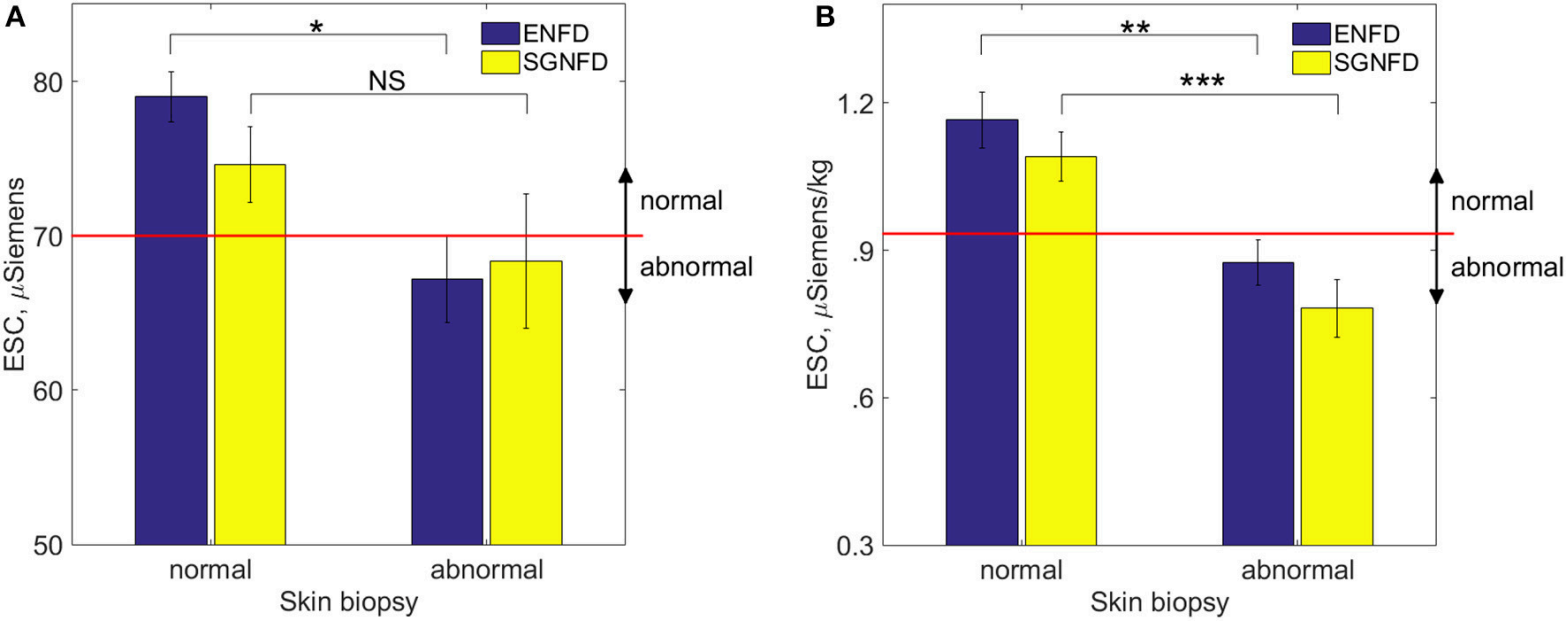
**Comparison of ESC (mean ± se) in subjects with normal and abnormal skin biopsies using unadjusted raw ESC (A) and ESC data adjusted for weight (B)**. Recommended threshold for normal range is ≥ 70 μSiemens **(A)** per the manufacturer of the device corresponding approximately to 0.93 μSiemens per kg on adjusted ESC data **(B)**. ^*^p < 0.04; NS, not significant; ^**^p < 0.0008; ^***^p < 0.0003. ESC, electrochemical skin conductance; SGNFD, sweat gland nerve fiber density; ENFD, epidermal nerve fiber density.

Figure 2 shows an example of ENFD and SGNFD from our cohort. Subjects with abnormal ENFD (*n* = 58) had reduced feet ESC (mean ± sd: 0.88 ± 0.35 μSiemens/kg) compared to subjects with normal ENFD (*n* = 23, ESC = 1.17 ± 0.27 μSiemens/kg, *p* < 0.0008 using Wilcoxon non-parametric test, Figure 3). Subjects who had abnormal SGNFD (*n* = 25) had reduced feet ESC (mean ± sd; 0.78 ± 0.3 μSiemens/kg) compared to subjects with normal SGNFD (*n* = 41, ESC = 1.09 ± 0.34 μSiemens/kg, *p* < 0.0003 using *t*-test, Figure 3).

**Figure 4:**
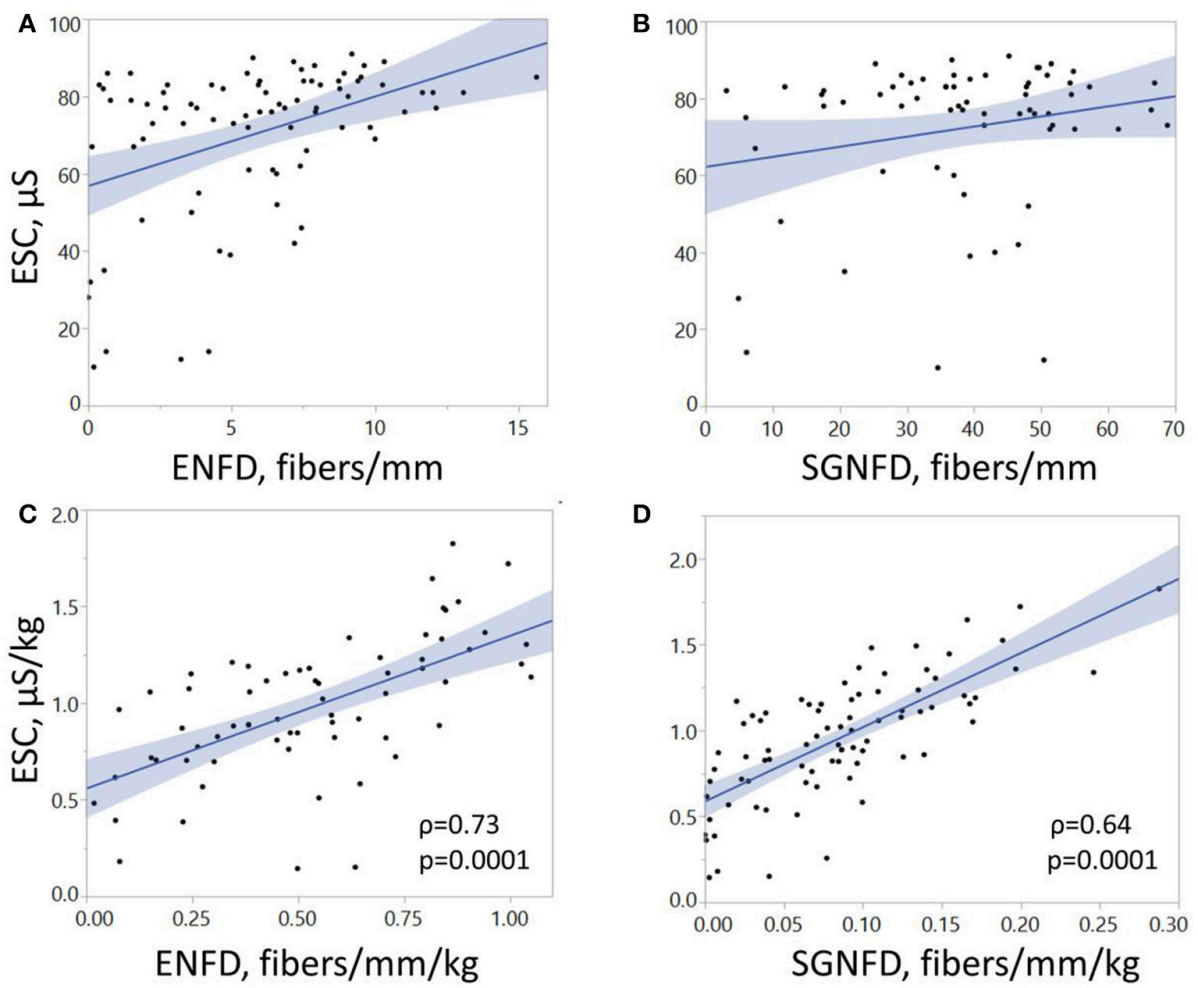
**Correlations of electrochemical skin conductance with ENFD and SGNFD**. Each dot represents one patient. Panels **(A,B)** show raw data, while panels **(C,D)** depict data adjusted for weight. ESC, electrochemical skin conductance; μS, microSiemens; ENFD, epidermal nerve fiber density; SGNFD, sweat gland nerve fiber density.

Feet ESC correlated with ENFD and SGNFD at the calf (Table 1, Figure 4). ESC did not correlate with QSART at the foot (ρ = 0.2, *p* < 0.07 both unadjusted and ρ = 0.2, *p* < 0.06 both adjusted). QSART at the foot did not correlate with ENFD (ρ = −0.008, *p* < 0.94 both unadjusted, ρ = 0.1, *p* < 0.37 both adjusted) or SGNFD (ρ = −0.14, *p* < 0.26 both unadjusted and ρ = 0.006, *p* < 0.95 both adjusted). ENFD correlated with SGNFD (ρ = 0.52, *p* < 0.0001, unadjusted data).

**Table 1:** **Pairwise correlations between adjusted ESC and measured variables**.

**Variable 1**	***P***	***p*-value**
SGNFD	ρ = 0.64, *n* = 66	=0.0001
ENFD	ρ = 0.73, *n* = 81	=0.0001
Hemoglobin A1c	ρ = −0.4, *n* = 39	=0.01
NPSI	−0.05	=0.67
Pain scale (0–10)	−0.02	=0.88
NTSS-6	−0.05	=0.67
SAS	0.17	=0.13

*ESC, electrochemical skin conductance at the feet adjusted for weight. Pearson correlation (r) and Spearman's rank correlation (ρ) coefficients have been used for data with normal and non-normal distributions, respectively. SGNFD, sweat gland nerve fiber density; ENFD, epidermal nerve fiber density; NPSI, the Neuropathic Pain Symptom Inventory; NTSS-6, the Neuropathy Total Symptom Score-6; SAS, the Survey of Autonomic Symptoms*.

ESC did not correlate with subjective pain and autonomic scales (Table 1). QSART at the foot did not correlate with the pain (0–10) scale (ρ = −0.07, *p* < 0.48), the Neuropathic Pain Symptom Inventory (ρ = −0.15, *p* < 0.17), the Neuropathy Total Symptom Score-6 (ρ = −0.18, *p* < 01), or the Survey of Autonomic Symptoms (ρ = −0.02, *p* < 0.84).

Feet ESC did correlated with hemoglobin A1C [ρ = −0.4, *p* = 0.01, number of subjects (*n*) = 39]. ESC did not correlate with folate (ρ = −0.09, *p* = 0.6, *n* = 31), B12 (ρ = 0.05, *p* = 0.78, *n* = 37) or TSH (ρ = 0.22, *p* = 0.15, *n* = 43).

LS models showed significant effects of ENFD (*p* = 0.001), but not of age (*p* = 0.25) or gender (*p* = 0.06) on ESC adjusted by weight. LS models showed significant effects of SGNFD (*p* = 0.001) and age (*p* = 0.0018) but not gender (*p* = 0.13) on ESC adjusted by weight.

**Figure 5:**
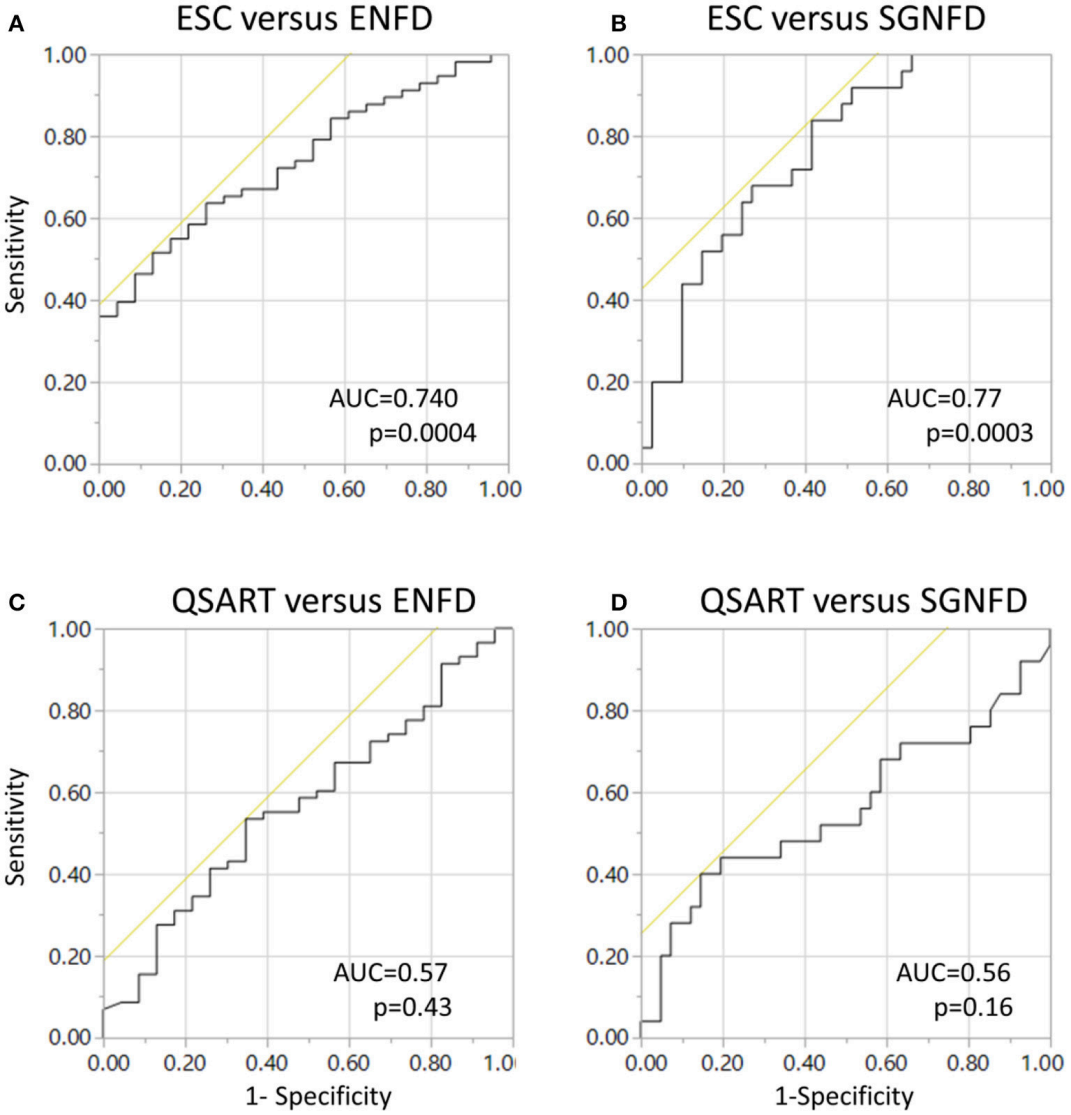
**Receiver operating characteristics (ROC) curves for ESC using ENFD (A) and SGNFD (B) as a reference**. Panels **(C,D)** show ROC of unadjusted QSART using ENFD and SGNFD, respectively. ROC = 0.6, *p* < 0.26 and ROC = 0.5, *p* < 0.46 for QSART adjusted for weight for ENFD and SGNFD, respectively. ENFD and SGNFD have been adjusted for weight in all calculations.

ROC curves (Figure 5) were significant for adjusted ESC using ENFD (Figure 5A) and SGNFD (Figure 5B) but not for QSART using ENFD (Figure 5C) or SGNFD (Figure 5D).

## Discussion

In this functional-pathological study, ESC was compared to nerve fiber densities obtained from skin biopsies. The main result of the study is that feet ESC is reduced in subjects with abnormal ENFD and SGNFD and that the reduction in ESC is proportional to the reduction of skin fibers. These findings indicate that reduced ESC may reflect axon loss. The correlation is remarkable considering the fact that ESC is measured at the soles of the feet while the biopsy was done at the calf. Ideally in a direct histologic-functional correlation both tests should be performed at the same location. However, both tests were implemented using recommended standards. ENFD and SGNFD have never been validated on the soles of the feet and ESC measurements are currently only possible at the soles and palms.

It can be argued that the significant correlations between ESC and skin biopsies could be driven by a few outliers as the unadjusted data implies. However, adjusted data have normal distribution and non-normal outliers were eliminated (compare Figures 1A, 3A, 4A with Figures 1B, 3B, 4B). Using adjusted ESC data may explain differences from previous studies. Our subjects were heavier (man BMI = 27.8 kg/m^2^) than those in previously reported studies (BMI < 25 kg/m^2^) (Vinik et al., 2015).

In our study, the ESC data were adjusted by weight and this adjustment resulted in normal ESC distribution indicating that ESC is affected by subjects' weight. Since the measurement occurs in the standing position, the weight-dependency could be due to the sensitivity of the stainless steel electrodes to pressure that is proportional to weight; alternatively, ESC depends on the size of the foot since heavier people may have larger feet. Both alternatives can be tested, by either eliminating differences in pressures by obtaining ESC in the supine position and/or by measuring the foot volume. Our data also suggest that the adjusted threshold for normative value is equal to 0.93 μSiemens/kg.

Previous studies using Sudoscan device were predominantly focused on diabetic patients (Smith et al., 2014; Vinik et al., 2015) while our study uses a more heterogenous population. ROC analysis for ESC obtained in our study (0.74, 0.767 for ENFD and SGNFD as a reference) were almost identical to those obtained in a previous study for ESC and ENFD (0.761, 0.752) using Utah Early Neuropathy Score as a reference (Smith et al., 2014). The most common diagnosis in our group was idiopathic SFN (59%) suggesting that our results are not linked to a particular disorder but may be applicable in general for evaluating small skin fibers.

ESC correlated better with sensory ENFD than with sudomotor SGNFD density. This might be surprising since it is thought that ESC measures chloride ion current produced by sweat glands. There are several possible explanations for this finding: (1) ESC response may also be dependent on the small sensory fibers; (2) Small sensory fibers degenerate along with the sudomotor fibers, hence the correlations are not causative; (3) SGNFD also measures in part small sensory fibers. A cardinal assumption made is that SGNFD evaluates sudomotor fibers. However, the marker used for fiber staining, namely PGP 9.5, is pan-axonal and it does not differentiate fiber subtypes. Although occasionally CGRP-positive sensory fibers can be detected within sweat glands, much denser innervation of sweat glands occurs from sudomotor sympathetic fibers (Wang et al., 2011). Hence it is unlikely that sensory innervation contributes significantly to SGNFD. Nevertheless, studies with enrollment of subjects with selective involvement of either small sensory or sudomotor fibers, for example subjects with congenital absence of sweat glands may clarify if ESC reflects the function of small sensory fibers, sudomotor fibers or both.

### Limitations of the study

Referral bias and a relatively small number of patients could affect results. In spite of our best efforts, hemoglobin A1C was obtained in only 39 subjects. Therefore although the significant correlation between ESC and hemoglobin A1C is promising, it needs to be confirmed by larger studies. Lack of correlation of ESC with hemoglobin A1C in previous studies can be explained by using unadjusted ESC data. In diabetic subjects ESC correlates with NCS (Selvarajah et al., 2015) which measure large fibers. Than a question arises if ESC may be influenced in part by function of large fibers. Since the current study did not evaluate NCS, the impact of large fibers on ESC should be addressed in future studies. Another limitation of the study is the use of 1 to 2 sweat glands for SGNFD and that no sweat glands were identified in 12 subjects. An increased yield could be achieved by using a larger diameter biopsy punch or deeper biopsies. However this increases the invasiveness of the procedure which may result in biopsy complications. Finally, it remains to be determined how accurately ESC can track changes in small nerve fibers longitudinally (Selvarajah et al., 2015).

## Conclusions

This study demonstrates a correlation between structural (ENFD and SGNFD) and functional (ESC) tests. The main strengths of our study are: (1) its prospective nature, (2) the favorable distribution of study subjects compared to typical SFN patient populations and (3) the effective blinding of the data processing since ENFD and SGNFD were analyzed in a separate institution.

Longitudinal studies may be better suited to assess the accuracy of ESC in the detection of axon loss.

## Author contributions

The author conceived and designed the study, performed experiments, analyzed the data and wrote the manuscript.

### Conflict of interest statement

The author declares that the research was conducted in the absence of any commercial or financial relationships that could be construed as a potential conflict of interest.

## Abbreviations

ESC: electrochemical skin conductance
ENFD: epidermal nerve fiber density
SGNFD: sweat gland nerve fiber density
HbA1c: Hemoglobin A1c
QSART: quantitative sudomotor axon reflex test
SFN: small fiber neuropathy
TSH: thyroid stimulating hormone.
